# A Novel Medical E-Nose Signal Analysis System

**DOI:** 10.3390/s17040402

**Published:** 2017-04-05

**Authors:** Lu Kou, David Zhang, Dongxu Liu

**Affiliations:** 1 Biometrics Research Center, Department of Computing, The Hong Kong Polytechnic University, Kowloon 999077, Hong Kong, China; cslkou@comp.polyu.edu.hk; 2 Department of Computer Science, Harbin Institute of Technology Shenzhen graduate school, Shenzhen 518055, China; liudongxu@alu.hit.edu.cn

**Keywords:** e-nose, chemical sensors, breath analysis, blood glucose level

## Abstract

It has been proven that certain biomarkers in people’s breath have a relationship with diseases and blood glucose levels (BGLs). As a result, it is possible to detect diseases and predict BGLs by analysis of breath samples captured by e-noses. In this paper, a novel optimized medical e-nose system specified for disease diagnosis and BGL prediction is proposed. A large-scale breath dataset has been collected using the proposed system. Experiments have been organized on the collected dataset and the experimental results have shown that the proposed system can well solve the problems of existing systems. The methods have effectively improved the classification accuracy.

## 1. Introduction 

Electronic noses, or e-noses, are devices that “smell” or detect odor. An e-nose consists of a mechanism for chemical detection, such as an array of electronic sensors, and a mechanism for processing. Different sensors respond differently to odor samples and transmit the signal to the processing module. By analyzing the signals, the components or characteristics of the samples can be distinguished. E-noses are now attracting more and more interest from researchers because of the wide range of applications [1], including drunk-driving testing, hazardous gas monitoring [2] and air quality monitoring [3,4,5]. Table 1 lists some of the e-noses manufacturers and the application of their products. These commercial e-noses always must provide some versatility in applications, such as coffee, wine, and fragrance identification for the sake of their marketing concerns. The versatility may in contrast improve the price and limit performance since their sensor selection must match broad applications. Therefore, it is a better choice to design a specific e-nose system for specific applications.

Medical applications comprise another important area of e-noses. They have been used in medicine for the diagnosis of renal disease [12,13], diabetes [14], lung cancer [15,16], arthritis [17], and asthma [18]. Although gas chromatography (GC) has been proven to be effective in breath diagnosis [19], using e-nose instead of GC [20] to analysis human breath is generally cheaper, faster, more portable, and easier to operate [21].

**Figure 1:**
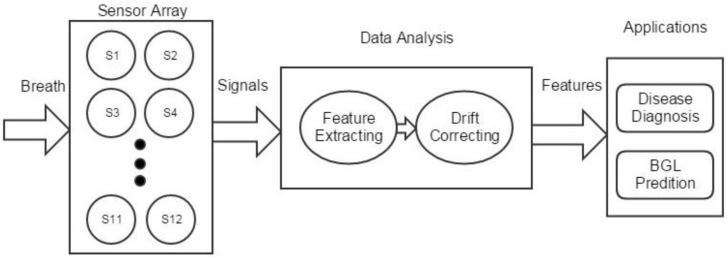
Global working flow of the system. BGL: blood glucose level.

However, most of the existing trials [22] on breath diagnosis only focus on very limited kinds of diseases. One possible reason might be the design of commercial e-noses for broad applications rather than for breath analysis specifically or that the specific designed devices only detect limited components [23]. Moreover, some of the fluctuations in breath samples were not well taken into account, such as humidity and the ratio of alveolar air. Furthermore, the numbers of samples in the experiments of previous studies are small. We thus designed a novel medical e-nose device for breath analysis with optimized structure and sensor arrays for the specific application in order to extend the applications in medicine. A breath analysis dataset was then collected by this e-nose. Experiments were organized on the collected dataset to evaluate the performance of the system in disease diagnosis and blood glucose level (BGL) classification. By analyzing the e-nose signals of human breath, it is possible for us to recognize the difference in contribution of the biomarkers so that certain diseases can be detected [24]. For example, acetone [25,26,27] as well as many other volatile organic compounds (VOCs) [28] in breath have already been proven to either have abnormal concentrations in diabetics or correlate with BGL. Moreover, in order to reduce the influence of device variety and time drift and make the system more robust, drift compensation methods should be introduced in the system before classification. Figure 1 gives a global working flow of the system.

## 2. Optimal System Design 

### 2.1. Sensor Array Selection

Human breath is largely composed of oxygen, carbon dioxide, water vapor and nitric oxide, and the rest is less than 100 ppm (parts per million) of mixture with over 500 kinds of components, including carbon monoxide, methane, hydrogen, acetone and numerous volatile organize compounds (VOCs) [21,29]. As the metabolic processes and partition from blood change with some diseases, the types and concentrations of components in human breath will also change. 

Nowadays, the concentration of some biomarkers in breath has been proven to be related with certain diseases. By selecting proper sensors that can respond to the components, it is possible to analyze a person’s breath odor and thus their health state. A few examples will further prove these points. The level of nitric oxide can be used as a diagnostic for asthma [30]. Patients with renal disease have higher concentrations of ammonia [31]. The concentration of VOCs, such as cyclododecatriene, benzoic acid, and benzene are much higher in lung cancer patients [32]. Table 2 lists the relationship between biomarkers and some typical diseases.

Among the breath biomarkers related with BGL, acetone is higher in concentration and easier for analysis. People with diabetes have insufficient insulin secretion or cannot effectively use their own insulin. As a result, it is difficult for glucose to enter the cells, leading to the rising of the BGL. On the other hand, because the cells cannot get enough energy, the liver will increase lipolysis and produce more ketones. Other biomarkers in lower concentrations include ethanol and methyl nitrite [36].

Taking the relationship between breath biomarkers and diseases into consideration, the sensors in the device should be sensitive to the VOCs, carbon dioxide, humidity, and temperature. Thus, a sensor array with 11 sensors is optimized for the purpose of detecting one’s breath. The sensor array includes six ordinary metal oxide semiconductor (MOS) sensors, three temperature modulated MOS sensors, a carbon dioxide sensor, and a temperature-humidity sensor. Specifically, the temperature-humidity sensor has two input channels for temperature and humidity respectively. As a result, there are, in total, 12 input channels. The model, manufacturer and function of the sensors are listed in Table 3. The suffix “-TM” indicates a temperature-modulated sensor.

### 2.2. Optimized System Structure

**Figure 2:**
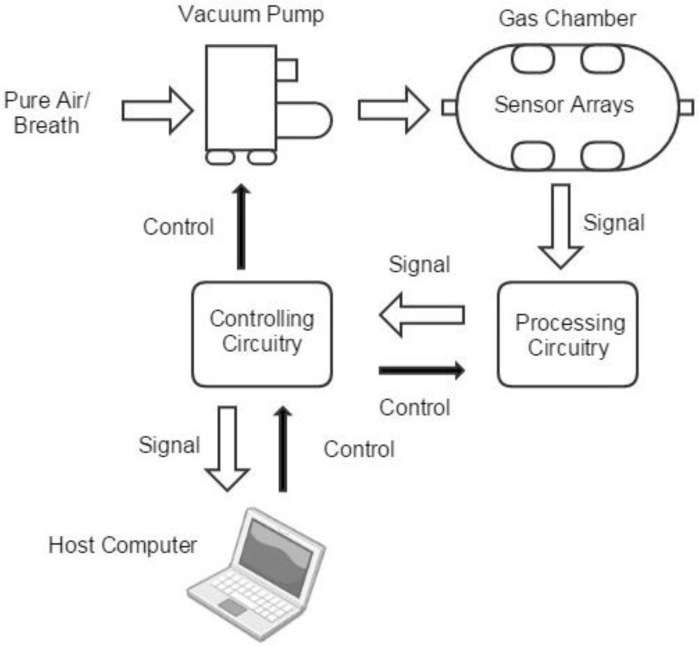
The frame of the e-nose system with five modules: the gas route, the sensor arrays, the signal processing circuitry, the controlling circuitry and the host computer.

Figure 2 shows the frame of the proposed e-nose system, including mainly five modules: the gas route, the sensor arrays, the signal processing circuitry, the controlling circuitry and the host computer.

**Figure 3:**
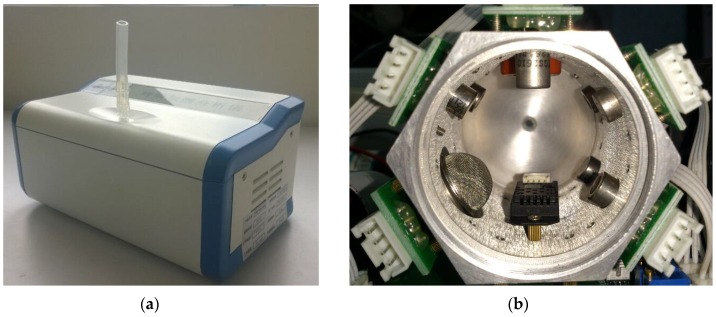
Snapshot of the device (**a**) and gas chamber (**b**). Sensors are embedded on its wall. Samples are injected to the chamber from the inlet hole at one end and pumped out through the outlet end.

The gas route of the device contains a vacuum pump and a gas chamber. Breath samples or air from outside was drawn and injected into the gas chamber. With the purpose of finding a balance between portable and effectiveness, the gas chamber is designed to be a column-shaped metal container with the capacity of 100ml. Sensors are embedded on the six facets, so that gases can flow smoothly. The gas concentration in the head space of each sensor is similar, and the size of the chamber can be kept relatively small, as shown in Figure 3. 

The resistances of the sensors change from *R*_0_ to *R_S_* when they are exposed to sampled gas. The output voltage is:

$$V_{Out}=\frac{1}{2}V_{CC}\left(1-\frac{R_{S}}{R_{0}}\right)$$

where *V_CC_* is the transient voltage crossing the sensor and *V_Out_* is the transient output voltages of the measurement circuits.

The origin sensors’ signals are magnified by a signal processing circuit. The signal processing circuit also filters high frequency noises. The controlling circuitry is used to control the pump and the processing circuitry, then it digitizes the processed signals and transmits them to a host computer for further processing. In order to take away the heat emitted by the sensors, a fan is set next to the gas chamber. The fundamental parameters of the system are summarized in Table 4.

### 2.3. Sampling Procedure

The temperature of the sensors goes up to a relatively stable level during use, which results in a change in baseline response of the sensors. Therefore, the device should be switched on for about 20 min until the baseline response shown on the host computer is stable. Besides, the devices should be calibrated every two weeks with 10 different kinds of standard gas samples to reduce the time drift. The standard gas samples include VOCs, H_2_, CO_2_, NH_3_ and healthy breath samples, with two different concentrations respectively.

Tedlar gas bags (600-mL) supplied by Beijing Safelab Technology Ltd. were used to collect breath samples. Subjects were asked to take a deep breath and exhale into a gas bag through a disposable mouthpiece and an airtight box filled with disposable hygroscopic material to absorb the water vapor. The gas bags also allowed those weak patients to exhale enough with more than one expiration. Then, the gas bag with the breath sample was plugged onto the preheated device connected to a computer. The measurement procedure was automatically controlled by the software in the computer. The measurement procedure was divided to four stages:

(1) Baseline stage (0–1 s): The baseline values of the sensors were recorded for future data preprocessing.

(2) Injection stage (1–8 s): The pump was ON. Breath was drawn from the gas bag to the gas chamber at a constant speed. The sensors’ signals started to respond to the injected breath.

(3) Reaction stage (9–64 s): The pump was OFF. The sensors continued reacting with the components in breath. The responses of the MOS sensors without temperature modulation (TM) reached their maximum values.

(4) Purge stage (64–144 s): The pump was ON again. Clean air was drawn in to clean the gas chamber for 80 s. The sensors’ responses gradually returned to their baselines. After the responses remained stable in their baselines, the device was ready for the measurement of the next sample.

**Figure 4:**
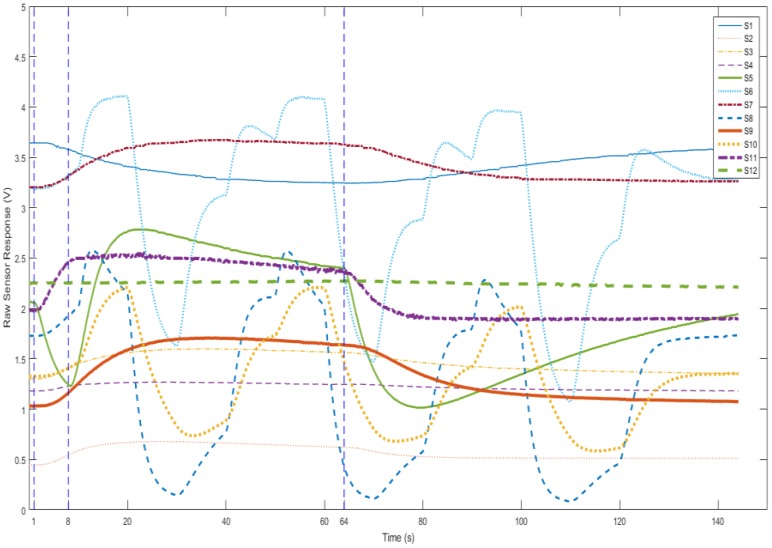
The four stages of measurement procedure.

Figure 4 shows the responses of the sensors (S1 to S12) in four stages. It can be seen that the responses keep stable in the baseline stage and start to change from the injection stage. Since the injection speed is 50 mL/s, 350 mL of the sample gases is pumped in. The first 250 mL directly go through the chamber to remove air in the chamber and the rest (100 mL) stays in the chamber for reacting. Each sensor reaches its highest response value at least once within the reaction stage. Finally, during the purge stage, the sample gas is cleaned by pure air and the responses return to baseline. The system does not require very high sampling frequency (chosen at 8 Hz). After the whole process, a digitized breath sample was represented by 12 response curves. Each response curve has 144 s × 8 Hz = 1152 data points. The samples were then used for further analysis.

## 3. Signal Analysis

### 3.1. Preprocessing

Before analyzing data, original signals should be preprocessed so as to be transformed into standard samples. Four steps were taken: faulty signal removal, de-noising, baseline manipulation and normalization.

A faulty signal is a common problem in devices with sensors. In our system, causes of faulty signals are complicated, and include misoperation, bad connection and device damage. In order to make the system more robust, these signals should be removed before analysis. 

De-noising aims to remove the noise from the original signals by utilizing a low-pass filter to remove the noise since the signal is mainly interfered by high-frequency noise.

The purpose of baseline manipulation is to compensate baseline drift. The baseline value is the average response in the baseline stage of each sensor. The value is then subtracted from the whole response curve to eliminate the interference of background noise of the sensors [37]. Assume that for each sensor transient of each sample, there are *k* dimensions, where *k* = 1, …, *N_k_*, and *b* dimensions in the baseline stage, where *b* = 1, …, *N_b_*. The response at time *t_k_* is denoted as $R\left(t_{k}\right)$. The baseline response is $B\left(t_{b}\right)$. Then baseline manipulation can be computed as:

$$R^{B}\left(t_{k}\right)=R\left(t_{k}\right)-\frac{1}{N_{b}}\overset{N_{b}}{\underset{t_{b}=1}{\sum }}B\left(t_{b}\right)$$

Normalization is used to compensate for sample-to-sample variations caused by analyte concentration. $R^{B}\left(t_{k}\right)$ is a sample after the baseline manipulation step, and the normalized response $R^{BN}\left(t_{k}\right)$ can be defined as:

$$R^{BN}\left(t_{k}\right)=\frac{R^{B}\left(t_{k}\right)}{\max\left(R^{B}\left(t_{k}\right)\right)}$$

### 3.2. Feature Extraction

To reduce the dimension of the origin features, principal component analysis (PCA) can be used. PCA projects high-dimensional data into a low-dimensional subspace while keeping most of the data variance.

Some low-dimensional geometric features can also be extracted from the origin response curves. Traditional features of gas sensors are their steady state responses. When a gas sensor is used to sense a gas sample, its response will reach a steady state in a few minutes. The steady state response has a close relationship with the concentration of the measured gas. Therefore, the 9D feature vector contains most of the information needed for disease screening.

However, additional useful information is carried in the transient responses [38]. Transient responses are often related to the change of gas flow (injection/purge) or temperature (for TM sensors). The feature set includes magnitude, difference, derivative, second derivative, integral, slope and phase features, as well as features in frequency domain such like fast Fourier transformation (FFT) and wavelet. The extracted features in both space domain and frequency domain are described in Table 5.

### 3.3. Drift Compensation

#### 3.3.1. Sensor Drift

Drift is a comment problem and challenging task for chemical sensors, which may influence the robustness of e-nose systems. The breath samples are collected by different devices in different time periods. On the one hand, because of the variations in of sensors and devices, the responses to the same signal source may not be different for different instruments. On the other, for the same device or same sensor, the stability also changes over time. 

Additionally, studies by Phillips M et al. [40] and Klaassen EM [41] also proved that there is relationship between age and breath biomarkers. Therefore, the influence of age should also be regarded as a drift factor. 

Widely used methods include algorithms based on variable standardization [42,43] and component correction principal component analysis (CC-PCA) [44] method. Moreover, Yan et al. proposed a drift correction auto-encoder (DCAE) [45].

Most of these methods require a set of predefined gas samples collected with each device and in each time period as transfer samples to provide mapping information between the source and the target domains. Nevertheless, collecting transfer samples may be a difficult job if there are not convenient predefined gases or the operators are not professional e-nose users. For this situation, as an unsupervised domain adaption method is proposed to correct the drifts with unlabeled data, the optimized maximum independence domain adaptation (MIDA) method will be used in the system for drift compensation.

#### 3.3.2. Optimized MIDA

Like transfer learning, domain adaption (DA) aims to solve the problem of transferring knowledge between domains with different distribution. Maximum MIDA proposed finding this latent subspace in which the samples and their domain features are maximally independent in the sense of Hilbert–Schmidt independence criterion (HSIC) [46].

HSIC measures the dependence between two sample sets $X,\;Y\in R^{d\times n}$:

$$\mathrm{HSIC}={\left(n-1\right)}^{-2}tr\left(K_{x}HK_{y}H\right),\;H=I-n^{-1}1_{n}1_{n}^{T}\in R^{n\times n}$$

We first define the domain features to describe the background information: the device label, the acquisition time and the age. Supposing there are $n_{dev}$ devices, the domain feature is then $d\in R^{3n_{dev}}$, and

$$d_{q}=\{\begin{array}{ll} 1, & q=3p-2\\ t, & q=3p-1\\ age, & q=3p\\ 0, & otherwise \end{array}$$

Suppose $X\in R^{m\times n}$ is the matrix of n samples containing both the training and the test samples. More importantly, we do not have to explicitly differentiate which domain a sample is from. A linear or nonlinear mapping function Φ can be used to map X to a new space. According to the kernel trick, the inner product of Φ(X) can be represented by the kernel matrix $K_{x}\;=\;\Phi {\left(X\right)}^{T}\Phi \left(X\right)$. Then, a projection matrix $\tilde{W}$ is applied to project Φ(X) to a subspace with dimension h, leading to the projected samples $Z\;=\Phi \left(X\right)\tilde{W}\in R^{h\times n}$. To express each projection direction as a linear combination of all samples in the space, $\tilde{W}=\Phi {\left(X\right)}^{T}W,\;W\in R^{n\times h}$ is the projection matrix to be actually learned. Thus, the projected samples are:

$$Z\;=\;\Phi \left(X\right)\Phi {\left(X\right)}^{T}W=K_{x}W$$

The kernel matrix $K_{z}=K_{x}WW^{T}K_{x}$.

On setting the matrix of the background feature as $D\in R^{n\times m_{d}}$, $m_{d}$ is the dimension of background feature. The linear kernel $K_{d}=DD^{T}$. On omitting the scaling factor in HSIC, the expression to be minimized is:

$$tr\left(K_{z}HK_{d}H\right)=tr\left(K_{x}WW^{T}K_{x}HK_{d}H\right)$$

In the domain adaption problem, the other goal is to preserve important properties of data, such as the variance, by maximizing the trace of the covariance matrix of the project samples.

$$\mathrm{cov}\left(Z\right)=\mathrm{cov}\left(K_{x}W\right)\;=\frac{1}{n}{\left(K_{x}W-\frac{1}{n}1_{n}1_{n}^{T}K_{x}W\right)}^{T}\left(K_{x}W-\frac{1}{n}1_{n}1_{n}^{T}K_{x}W\right)=W^{T}K_{x}HK_{x}W$$

Thus, the learning problem then becomes:

$$\underset{W}{\max}-tr\left(W^{T}K_{x}HK_{d}HK_{x}W\right)+\mu tr\left(W^{T}K_{x}HK_{x}W\right)\;s.t.\;W^{T}W=I$$

Using the Lagrangian multiplier method, we can find that W is the eigenvectors of $K_{x}\left(-HK_{d}H+\mu H\right)K_{x}$ corresponding to the h largest eigenvalues.

## 4. Experiments

### 4.1. Breath Dataset

To evaluate the performance of our device, a large-scale breath dataset was collected. We cooperated with Guangzhou Hospital of Traditional Chinese Medicine and collected data from inpatient volunteers. Two devices with same model were used for data collection. Patients were asked to rinse their mouth with medical mouthwash and not to use fragrance. The devices were placed in a well-ventilated room without the interruption of medical alcohol or odor of traditional Chinese medicine. For each sample, we first collected the patient’s breath and recorded the signals. The diagnosis was then given by an authoritative doctor as the classification labels. Moreover, some biochemical indicators were also collected, such as blood glucose, blood pressure and blood lipids. Finally, in this dataset, there were in total over 10,000 samples of 47 classes, including 1491 healthy samples and samples of 46 different kinds of diseases. In this paper, a subset of healthy samples and samples for six kinds of diseases were selected for experiments, including breast disease, cardiopathy, diabetes, lung disease, kidney disease and gastritis. Table 6 shows the number of each class used in selected subset. 

All the samples were collected from hospitals in Guangzhou. However, since most of the healthy samples were provided by medically-examined young people while disease samples were from elder patients, it is difficult to make age-matched subsets, which is a limitation of this dataset. Operations will be performed to reduce the impact of age. 

### 4.2. Disease Diagnosis

To check the performance of the system, six binary-classification tasks were performed to detect samples with one of the diseases from the healthy ones. 

**Figure 5:**
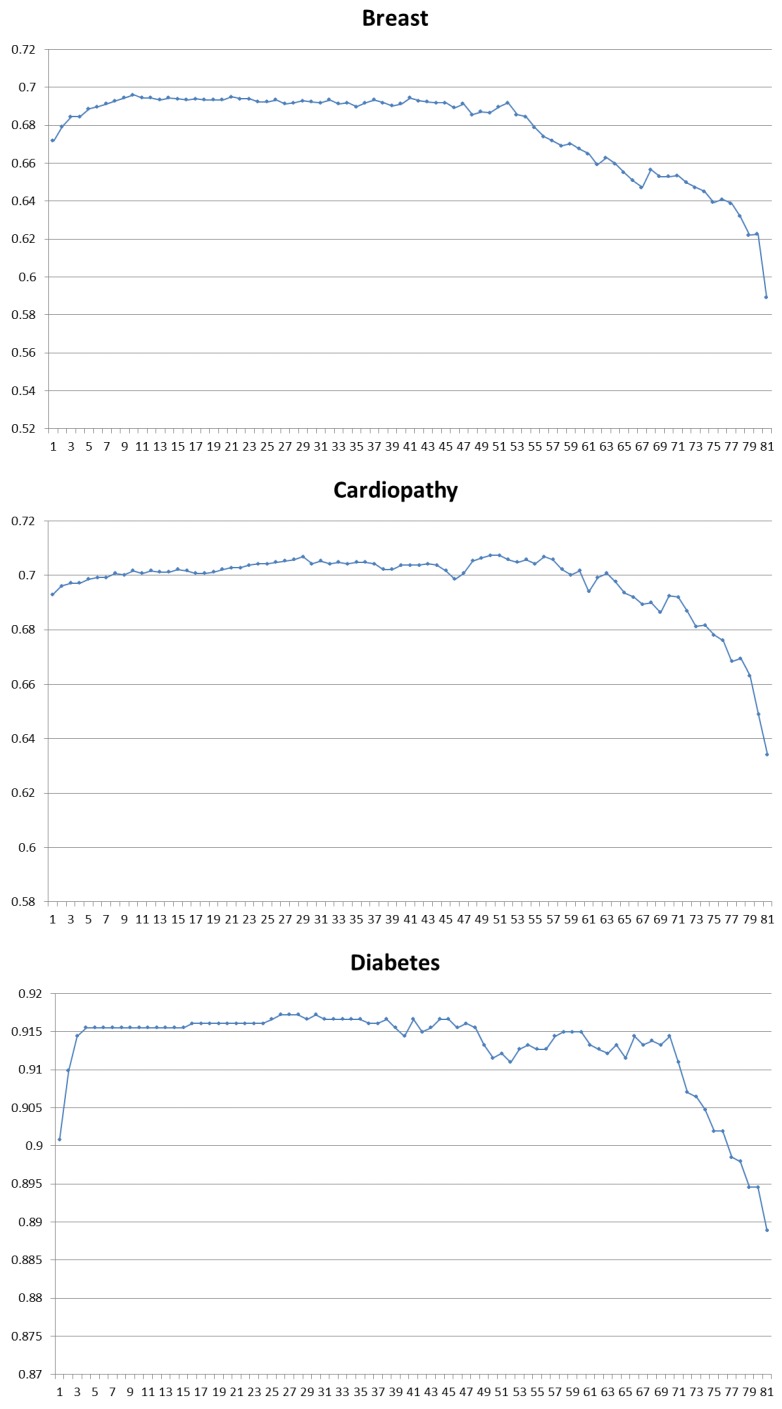

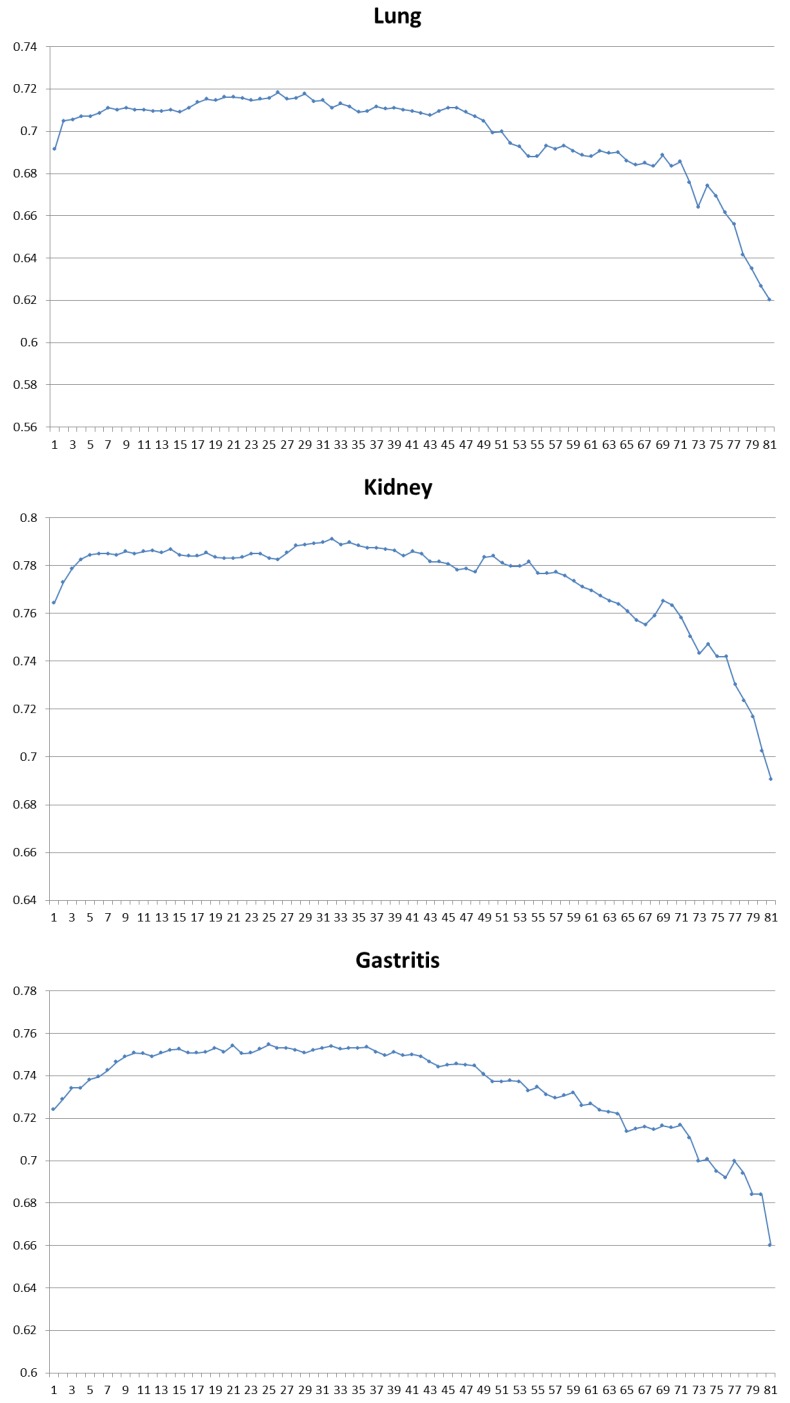
Forward selection result of six binary-classification tasks. For each graph, the horizontal axis is the number of features used and the vertical axis is the classification accuracy.

For each class, the first 50 samples collected by the first device were chosen as the labeled training sets and the rest were test samples. Logistic regression method was adopted as the classifier after drift compensation-optimized MIDA. Sequential forward selection (SFS) method was used to optimize the features. SFS method is a greedy strategy. In each iteration, one feature was selected from all features that could achieve the best classification accuracy together with the features already selected. Figure 5 shows the results of forward selection in different disease diagnosis tasks.

In Table 7, we conclude the best combination of features and sensors selected for each task. It can be find that Wavelet, MaxMag, Slope and Phase features contribute most in all the tasks. MeanMag feature and Integral feature are also discriminating in detecting cardiopathy, lung disease and gastritis. Derivative features only show its importance in tasks of breast disease and gastritis. Other features did not improve the performance of the system. On the other hand, the sensors that contribute most in each task to meet the relationship between diseases and breath biomarkers are listed in Table 2 and Table 3.

### 4.3. BGL Classification

BGL prediction is another application of the system. In the collected dataset, the blood glucose levels are given by blood glucose meters in hospital. Since the blood glucose meters may already have a ±10% to ±25% error, the final error rate will be further accumulated if we use these samples for regression. As a result, instead of regression experiments, we grouped the samples into different classes based on the BGL ranges and performed the BGL classification experiments on the datasets.

According to Chinese diabetes control criterion [47], the dataset was divided into four parts based on BGL. The BGL range and sample number of each class are listed in Table 8. Because of the detection error of the meters, samples within ±0.2 mmol/L of the thresholds were not selected as learning samples to improve the robust of the classification methods. We used a random forest (RF) method for the triplet-classification task.

The classification result and optimal features can be seen in Table 9. It can be seen that magnitude features (MaxMag, MeanMag and DownSampleMag), slope features and integral features are the most important features for BGL classification, while the most useful sensors include TGS2602-TM, TGS2602, TGS826, TGS822, QS01 and TGS2610D.

## 5. Conclusions

This paper presented a novel medical e-nose system that specified on disease diagnosis and BGL prediction. The scientific basis, structure, optimizing strategies, sensor arrays, sampling procedure and signal preprocessing methods were introduced, as well as a large-scale medical dataset collected by the system. In order to better correct the drifts, an optimized domain adaption method was adopted in the system. Experiments were taken on the new collected datasets to evaluate the performance of both the system and the methods.

The experimental results showed that better accuracy can be achieved by an optimal combination of features and sensors for different tasks. Wavelet, MaxMag, Slope and Phase features are most significant in most of the disease diagnosis tasks, while different sensors contribute differently based on the relationship of diseases and biomarkers. The BGL classification tests also produced a satisfactory output. However, it is still possible to further improve the performance and extend the applications. Mainly multi-feature and multi-classification methods will be investigated in future work. Neural networks will also be introduced to the system to discover the deeper relationship between the signals and human states.

## Figures and Tables

**Table 1:** The developed e-nose products.

Manufacturer	Product	Application
The eNose Company, Rotterdam, The Netherlands	AEONOSE [6]	Medical
Airsense Analytics GmnH, Schwerin, Germany	PEN3 [7]	Food, wine, matierial, enviroment, medical
Alpha-Mos, Toulouse, France	HERACLES [8]	Food, material, process management
Sensigent, Baldwin Park, CA, USA	Cyranose 320 [9]	medical, materials identification, food
Electronic Sensor Technology Inc., Newbury Park, CA, USA	Z-Nose [10]	Investigation, food, enviroment, medical
Owlstone Inc., Cambridge, UK	LONESTAR [11]	Food, materials, industry

**Table 2:** Breath biomarkers and related diseases.

Diseases	Breath Biomarkers
diabetes [24]	acetone
renal disease [31]	ammonia
heart disease [33]	propane
lung cancer [32]	benzene,1,1-oxybis-, 1,1-biphenyl,2, 2-diethyl, furan,2,5-dimethyl-, etc.
breast cancer [34]	nonane, tridecane, 5-methyl, undecane, 3-methyl, etc.
digestive system disease [35]	hydrogen

**Table 3:** Summary of the sensor array VOCs: volatile organic compounds; ppm: parts per million; TM: temperature-modulated.

Channel	Model	Manufacturer	Function	Sensitivities (ppm)
1	TGS4161	Figaro Inc., Osaka, Japan	CO_2_	350–10,000
2	TGS826	Figaro Inc., Osaka, Japan	VOCs, NH_3_	30–5000
3	QS01	FIS Inc., Hyogo, Japan	VOCs, H_2_, CO	1–1000
4	TGS2610D	Figaro Inc., Osaka, Japan	H_2_, VOCs	500–10,000
5	TGS822	Figaro Inc., Osaka, Japan	VOCs, H_2_, CO	50–5000
6	TGS2602-TM	Figaro Inc., Osaka, Japan	VOCs, NH_3_, H_2_S	1–30
7	TGS2602	Figaro Inc., Osaka, Japan	VOCs, NH_3_, H_2_S	1–30
8	TGS2600-TM	Figaro Inc., Osaka, Japan	H_2_, VOCs, CO	1–100
9	TGS2603	Figaro Inc., Osaka, Japan	NH_3_, H_2_S	1–10
10	TGS2620-TM	Figaro Inc., Osaka, Japan	VOCs, H_2_	50–5000
11	HTG3515CH	Humirel Inc., Toulouse, France	Temperature	
12			Humidity	

**Table 4:** Fundamental Parameters of the System.

Parameters	Specifications
Device size	22 × 15 × 11 cm
Working temperature	25 ± 10 °C
Gas chamber volume	600 mL
Injection rate	50 mL/s
Sampling frequency	8 Hz
Sampling time	144 s
Working voltage	5 V
Working voltage for temperature modulated sensors	3–7 V cycle
Resolution of the Analog-to-Digital Converter (ADC)	12 bit

**Table 5:** Summary of the transient features. PCA: principal component analysis.

Feature		Characteristics
Spacial	PCA	Reduced dimension of the origin features with PCA method.
	Magnitude	Down-sampled values of the curve’s magnitude M. The maximum magnitude. Down-sampled values of the normalized magnitude M/max (M). Mean values of the magnitude.
	Derivative	Down-sampled values of the curve’s derivative D. The maximum and minimum derivative.
	Second derivative	The maximum and minimum second derivative in both the injection and purge stage.
	Integral	The integral of the five intervals of the curve; the intervals are the same with the difference feature.
	Slope	The slope of the five intervals of the curve; the intervals are the same with the difference feature.
	Phase Feature	The phase feature is proposed in [39]. First, the response is transformed to the phase space, which is spanned by its magnitude and derivative. Then, the phase features are defined as $\int_{M\left(t_{i}\right)}^{M\left(t_{i+1}\right)}DdM$
Frequency	FFT	Fast Fourier tranformation
	Wavelet	Wavelet transformation

**Table 6:** Number of samples in each class.

Class	Number
Healthy	1291
Diabetes	491
Kidney disease	398
Cardiopathy	537
Lung disease	376
Breast disease	527
Gastritis	241

**Table 7:** Selected features and sensors, and the sensitivity (SEN), specificity (SPE) and accuracy (ACC) for each task.

Task	Features and Sensors		SEN	SPE	ACC
Diabetes	Wavelet of TGS2602 Phase of TGS2602-TM	Wavelet of TGS2610D MaxMag of TGS826	0.8815	0.9495	0.9155
Kidney Disease	Wavelet of TGS2602 Slope of TGS2602-TM Phase of TGS826	Wavelet of TGS2600-TM Slope of TGS2620-TM Phase of TGS2610D	0.7002	0.8698	0.7850
Cardiopathy	Wavelet of TGS822 MeanMag of TGS2603 Integral of TGS2602 MaxMag of TGS2603 Phase of TGS2610D	Integral of TGS826 Slope of TGS822 Slope of TGS826 Phase of TGS826 Integral of TGS2610D	0.7433	0.7125	0.7279
Lung Disease	Wavelet of QS01 Slope of TGS2603 Integral of TGS2602 Phase of TGS2620-TM MaxMag of TGS2602 MeanMag of TGS2610D MaxMag of TGS2603 Integral of QS01 MaxMag of TGS2602-TM Phase of TGS826 Phase of TGS2602	MeanMag of QS01 Slope of QS01 Integral of TGS826 Slope of TGS826 Integral of TGS2610D MeanMag of TGS2603 MeanMag of TGS2602-TM MeanMag of TGS2602 MaxMag of TGS826 Phase of QS01	0.7117	0.7209	0.7163
Breast Disease	Wavelet of TGS826 MaxMag of TGS2602 Derivative of TGS2620-TM Phase of TGS2600-TM MeanMag of TGS2602-TM	MaxMag of TGS822 Derivative of TGS2610D MaxMag of TGS2603 MeanMag of TGS2600-TM Phase of TGS2620-TM	0.6321	0.7599	0.6960
Gastritis	Wavelet of TGS822 Integral of TGS2603 Phase of TGS2602-TM Wavelet of TGS2620-TM MaxMag of TGS2602-TM	Slope of TGS2600-TM MaxMag of TGS2620-TM Integral of QS01 Derivative of TGS2610D Slope of TGS2603	0.6436	0.8582	0.7509

**Table 8:** Number of samples in each class of blood glucose levels (BGL).

Class	BGL (mmol/L)	Number
Normal	Lower than 6.1	1851
Impaired glucose tolerance	6.1–7.11	168
Hyperglycemia	Higher than 7.11	241

**Table 9:** Selected sensors and features for BGL classification.

Features and Sensors		Accuracy
MaxMag of TGS2602-TM	MaxMag of TGS2602	0.7778
MeanMag of TGS2602-TM	DownSample of TGS826	
DownSample of QS01 DownSample of TGS822 Slope of QS01	DownSample of TGS2610D Slope of TGS826 Integral of QS01	
